# The Association Between Social Jetlag and Glycemic Control in Diabetic Patients at King Saud University Medical City

**DOI:** 10.7759/cureus.9215

**Published:** 2020-07-15

**Authors:** Abdulaziz Alabdulkarim, Omar Alayed, Omar Aloraini, Mohammed Almozini, Khalifah Aldawsari, Yasir Z Bin Khathlan

**Affiliations:** 1 Medicine, King Khalid University Hospital, Riyadh, SAU; 2 Internal Medicine, King Khalid University Hospital, Riyadh, SAU; 3 Medicine, King Saud University, Riyadh, SAU

**Keywords:** social jetlag, diabetes, glycemic control, biological clock

## Abstract

Social jetlag (SJL) has been linked to many cardiovascular and metabolic diseases, as it disturbs the circadian rhythm. In this study, we analyzed the impact of SJL on glycemic control. To our knowledge, this was the first study that discussed the issue of SJL, and we explored the prevalence of SJL in the studied population. A case-control study matched by age and gender was conducted among 511 subjects. Control group subjects were diabetic with HbA1c levels of <7.5%, while our cases were diabetic with HbA1c levels of 7.5% or more. We used the Munich Chronotype Questionnaire (MCTQ) to assess SJL among the participants. Based on our findings, SJL status was similar for both cases and control participants, which indicates that there is no significant association between SJL and HbA1c levels (p=0.394). The prevalence of SJL in the studied population was 58.4%. Further studies are required to obtain a more precise estimation of sleep duration and SJL, and they should focus on SJL and its related problems.

## Introduction

The master circadian clock is located in the suprachiasmatic nuclei of the hypothalamus. It plays a significant role in controlling the circadian system and regulating the daily rhythms of sleep and waking and various metabolic outputs, such as feeding behaviors, peripheral tissue metabolism, and hormone secretion [1]. In modern societies, people with 24-hour access to artificial light often engage in activities that are timed inappropriately relative to their endogenous circadian rhythms. This disturbance in timing is called "circadian misalignment," and it has been linked to many cardiovascular and metabolic diseases [2]. The degree of disturbance depends on the person's "chronotype" [3]. Late chronotypes have been associated with a high degree of disturbance between social rhythms and the circadian clock. Chronic misalignment between one's circadian rhythm and the social clock is called social jetlag (SJL) [3]. A study has shown that people who live in modern societies have mild circadian disturbance, especially during work or school days, as they follow social rhythms that are imposed on them, such as school schedules, family meetings, and social obligations [3]. Another study involving 65,000 participants has demonstrated that 69% of the population experiences at least one hour of SJL [4].

Diabetes mellitus is a condition primarily characterized by hyperglycemia, and it leads to a risk of microvascular and macrovascular complications [5]. A recent systematic review demonstrated that diabetes prevalence in Saudi Arabia in 2016 was 32.8%, and it was projected to reach 45.4% in 2030 [6]. Recent studies have shown a correlation between sleep disturbance and the development of endocrine disorders, especially diabetes mellitus [2,7,8]. Poor sleep quality, lack of sleep, and SJL induce hyperglycemia and may also lead to worsening of glycemic control in diabetic patients [8,9]. A cohort study has reported that longer SJL would increase the incidence of diabetes and unfavorable metabolic symptoms [10]. Diabetic patients with SJL of over 30 minutes were predisposed to have higher HbA1c levels than those with SJL of under 30 minutes [11]. This study aimed to evaluate SJL and its association with glycemic control.

## Materials and methods

A case-control study matched by age and gender was conducted among 511 subjects. The cases and controls were selected from the medical charts of King Saud University Medical City (KSUMC) in Riyadh, Saudi Arabia, between January 2017 and April 2017. We included diabetic patients who had done an HbA1c test within the last three months (433) and excluded those who had not (78). All participants were interviewed by telephone, and they agreed to fill out a questionnaire. The case and control participants were matched by gender; in the control group, there were 110 males and 104 females, while in the case group, there were 110 males and 107 females. The mean age in the control group was 45.56 years, and the mean age in the case group was 47.58 years. Privacy and confidentiality were maintained during data collection. We used the self-administered Munich Chronotype Questionnaire (MCTQ) to assess SJL [12]. Since the original questionnaire was in English, it was translated into Arabic by a professional bilingual speaker; then, we gave the Arabic form to another professional bilingual speaker to translate it back into English. After comparing them, the authors concluded that there were no differences in meaning between the two versions.

A diabetes mellitus patient is defined as any subject who has been previously diagnosed as diabetic and is currently using anti-diabetic medications, or any subject who is found to have an HbA1c level of ≥6.5% as per WHO and American Diabetes Association [13]. The case group consisted of participants with uncontrolled glycemia who used anti-diabetic medications with HbA1C levels of ≥7.5% per WHO and American Diabetes Association [13]. The control group comprised glycemia-controlled participants who had HbA1c levels between 6.5% and 7.5% [13]. SJL is defined as the conflict between the internal clock and external clock, measured by the absolute difference between mid-sleep on work-days and mid-sleep on free days [3]. To calculate the sample size, we could not find any relevant material in the published literature about the association between SJL and glycemic control. Hence, we used an odds ratio of 2, estimated the prevalence of SJL among patients with controlled diabetes at 69% [4], and assumed a correlation of 0.1 between a case and control exposures for matched pairs. Thus, for 80% power, 95% confidence level, and 1:1 ratios, we needed a total sample of 382, i.e., 190 cases and 190 controls; then we added 15% more for response rate (440). The sample size was calculated using the statistical package R version 3.2.2. This study was approved by the Institutional Review Board of the College of Medicine at King Saud University (KSU) in 2017. Categorical data were summarized with absolute numbers and percentages, whereas continuous data were summarized as means and standard deviations (SDs) or medians and interquartile ranges (IQRs). A comparison between groups for categorical variables was made using the Chi-squared test or Fisher's exact test, whereas, for continuous data, the Student's t-test or the Mann-Whitney U-test were used. All the analyses were performed using SAS version 9.2 (SAS Institute, Inc, Cary, NC).

## Results

**Figure 1:**
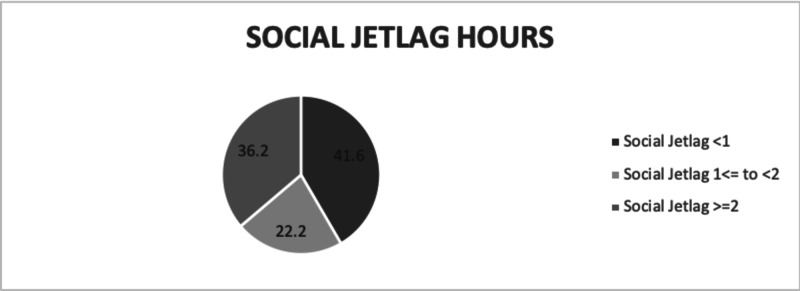
Classification of participants into groups based on SJL hours The darkest color represents people with SJL of less than one hour (41.6%). The gray shade represents people with SJL of one hour to less than two hours (36.2%). The light gray color represents people with two hours or more of SJL (22.2%) SJL: social jetlag

Our study included 511 participants. The mean age of the participants was 47.6 years, and 51.0% of them were male, according to the filled-in MCTQ. On average, our participants were obese (BMI=31.42), with a mean weight of 84 kg. About 61% of them were regularly employed; only 34 reported working more than five days a week, and only 24 had performed shift work in the three months prior to filling out the questionnaire. Of note, 58.4% of the studied population had one hour or more of SJL; about two-thirds of them had two hours or more of SJL, with a mean sleeping time of 6.7 hours on workdays and 7.85 hours on free days. The mean HbA1c level in our population was 7.98%, with a standard deviation of 1.9. Table 1 shows a summary of each parameter related to our study population. In Table 2, a comparison is made between diabetic controlled (control group) and diabetic uncontrolled (case group) participants using certain variables. There were 433 participants who had done the HbA1c test in the three months prior to filling out the questionnaire. The results were similar between the cases and controls, except for drinking coffee and taking sleep medications. The SJL status was almost similar among both controlled and uncontrolled patients, which indicates that there is no significant association between SJL and HbA1c (p=0.394). In a comparison between the SJL group and the non-SJL group (Table 3), we found that regular work led to SJL; an inverse relationship was found between working for more than five days and SJL. The use of an alarm clock during workdays was significantly associated with SJL. A total of 143 participants were found to be consuming caffeine, and 106 among them were found to have SJL (p<0.001). We subdivided the population into three groups: the first group (41.6%) consisted of participants without SJL and less than one-hour difference between sleeping hours on free days and those on workdays; the second group (36.2%) comprised participants with SJL of one hour to less than two hours; the third group (22.2%) had participants with two hours or more of SJL (Figure 1).

**Figure 2:**
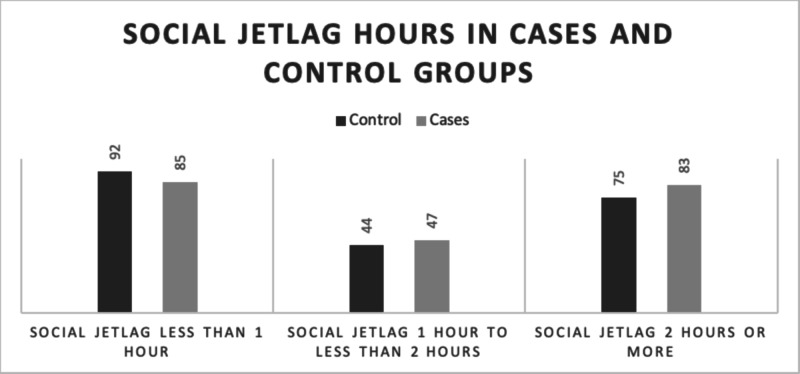
Number of participants in each SJL group from case and control populations The group with SJL of less than one hour had 92 participants from the control group and 85 from the case group. The group with SJL of one hour to less than two hours included 44 participants from the control group and 47 from the case group. Some of the participants had SJL of two hours or more, and this group had 75 from the control group and 83 from the case group SJL: social jetlag

All three groups showed almost similar results in the comparison between controlled type 2 diabetic patients and the uncontrolled type 2 diabetic patients. However, as shown in Figure 2, the numbers of case and control participants who had SJL for less than one hour were 85 and 92, respectively. The number of people with SJL for one hour to less than two hours was 44 in the control group and 47 in the case group. Some of the participants also developed SJL of two hours or more: 75 in the control group and 83 in the case group. Regarding using a stimulant, about 38% of the participants drank four or more cups of coffee, and about 81% of them drank it on a daily basis; 403 participants drank tea, and 295 of them consumed it daily. Only 241 were asked about smoking, and 38 confessed to being smokers; 270 were not asked, and most of them were female (we observed that the question was annoying them since it was socially inappropriate). Oddly, we found a direct relationship between SJL and the time it took to arrive at the workplace or home.

**Table 1:** Characteristics of the study population (N=511) SJL: social jetlag; WD: workday; FD: free day; BMI: body mass index; HbA1c: glycated hemoglobin; DM: diabetes mellitus; SD: standard deviation

Variable	Level	Value	%
Gender	Male	258	51.0
Group	Controlled DM	215	49.7
SJL status	SJL	292	58.4
Sleeping hours on workdays	Mean	6.8	-
Sleeping hours on free days	Mean	7.85	-
Regular work	Yes	311	61.1
Worked days	≤5 days	406	92.3
Alarm clock (WD)	Yes	296	57.9
I wake up before the alarm (WD)	Yes	121	39.2
Alarm clock (FD)	Yes	129	25.7
I freely choose my sleep time (FD)	Yes	152	31.0
I had shift work in the last 3 months	Yes	24	8.2
Flexibility of my work	Very flexible	91	34.0
	Little flexible	103	38.4
	Rather flexible	58	21.6
	Not very flexible	16	6.0
I travel to work in	Vehicles	256	84.2
	Foot/bike, etc.	16	5.3
	I work from home	32	10.5
Smoking	Yes	38	15.8
Coffee	<4 cups	305	62.1
Tea	Yes	403	84.0
Caffeinated drinks	Yes	143	38.5
Sleep medications	Yes	13	4.0
Age, years	Mean	47.64	-
	SD	12.23	-
Height, cm	Mean	163.61	-
	SD	9.77	-
Weight, kg	Mean	84.20	-
	SD	20.33	-
BMI, kg/m^2^	Mean	31.42	-
	SD	6.90	-
HbA1c, %	Mean	7.98	-
	SD	1.90	-
Working time hours	Mean	6.64	-
	SD	2.19	-

**Table 2:** Comparison between controlled diabetic (control group; n=215) and uncontrolled diabetic (case group; n=218) participants SJL: social jetlag; BMI: body mass index; HbA1c: glycated hemoglobin; DM: diabetes mellitus; SD: standard deviation

Covariate	Level	Controlled DM	Uncontrolled DM	Parametric p-value
Gender	Male	110 (50)	110 (50)	0.883
	Female	104 (49.29)	107 (50.71)	
SJL Status	SJL	119 (47.79)	130 (52.21)	0.394
	No SJL	92 (51.98)	85 (48.02)	
SJL hours	No SJL: SJL of <1 hour	92 (51.98)	85 (48.02)	0.69
	SJL of ≥1 to <2 hours	44 (48.35)	47 (51.65)	
	SJL of ≥2 hours	75 (47.47)	83 (52.53)	
Regular work	Yes	135 (50.94)	130 (49.06)	0.538
	No	80 (47.9)	87 (52.1)	
Worked days	≤5 days	166 (48.4)	177 (51.6)	0.117
	>5 days	19 (63.33)	11 (36.67)	
Smoking	Yes	19 (57.58)	14 (42.42)	0.292
	No	76 (47.5)	84 (52.5)	
Coffee	<4 cups	139 (57.2)	104 (42.8)	<0.001
	≥4 cups	64 (37.65)	106 (62.35)	
Coffee frequency	Daily	101 (57.39)	75 (42.61)	0.19
	Weekly	20 (52.63)	18 (47.37)	
	Monthly	4 (100)	0 (0)	
Black tea	Yes	172 (50.89)	166 (49.11)	0.071
	No	26 (38.81)	41 (61.19)	
Caffeinated drinks	Yes	60 (48.39)	64 (51.61)	0.642
	No	94 (51.09)	90 (48.91)	
Sleep medications	Yes	2 (18.18)	9 (81.82)	0.039
	No	128 (50)	128 (50)	
Age, years	N	210	218	0.083
	Mean	45.56	47.58	
	SD	11.3	12.72	
BMI, kg/m^2^	N	103	104	0.267
	Mean	31.03	32.12	
	SD	5.89	8.04	
HbA1c, %	N	215	218	<0.001
	Mean	6.57	9.37	
	SD	0.59	1.71	

**Table 3:** Comparison between SJL and non-SJL groups SJL: social jetlag; WD: workday; FD: free day; BMI: body mass index; HbA1c: glycated hemoglobin; DM: diabetes mellitus; SD: standard deviation

Covariate	Level		No SJL: SJL of <1 hour (n=208)	SJL of ≥1 to <2 hours (n=111)	SJL of ≥2 hours (n=181)	Parametric p-value		
Gender	Male	96 (37.65)		65 (25.49)	94 (36.86)	0.125		
	Female	108 (45)		45 (18.75)	87 (36.25)			
Group	Controlled DM	92 (43.6)		44 (20.85)	75 (35.55)	0.69		
	Uncontrolled DM	85 (39.53)		47 (21.86)	83 (38.6)			
Regular work	Yes	97 (31.7)		72 (23.53)	137 (44.77)	<0.001		
	No	109 (56.77)		39 (20.31)	44 (22.92)			
Worked day	≤5 days	153 (38.35)		85 (21.3)	161 (40.35)	0.107		
	>5 days	15 (46.88)		10 (31.25)	7 (21.88)			
Alarm clock (WD)	Yes	85 (29.21)		64 (21.99)	142 (48.8)	<0.001		
	No	123 (58.85)		47 (22.49)	39 (18.66)			
I wake up before the alarm (WD)	Yes	51 (42.86)		25 (21.01)	43 (36.13)	<0.001		
	No	41 (22.16)		41 (22.16)	103 (55.68)			
Alarm clock (FD)	Yes	54 (42.19)		23 (17.97)	51 (39.84)	0.354		
	No	150 (40.76)		88 (23.91)	130 (35.33)			
I freely choose my sleep time (FD)	Yes	45 (29.8)		36 (23.84)	70 (46.36)	0.002		
	No	155 (46.41)		70 (20.96)	109 (32.63)			
I had shift work in the last 3 months	Yes	5 (20.83)		6 (25)	13 (54.17)	0.729		
	No	75 (28.41)		61 (23.11)	128 (48.48)			
Flexibility of my work	Very flexible	25 (28.09)		21 (23.6)	43 (48.31)	0.18		
	Little flexible	35 (33.98)		23 (22.33)	45 (43.69)			
	Rather flexible	11 (18.97)		13 (22.41)	34 (58.62)			
	Not very flexible	2 (12.5)		2 (12.5)	12 (75)			
I travel to work in	Vehicles	66 (25.98)		55 (21.65)	133 (52.36)	0.003		
	Foot/bike, etc.	6 (37.5)		4 (25)	6 (37.5)			
	I work from home	12 (42.86)		12 (42.86)	4 (14.29)			
Smoking	Yes	15 (39.47)		10 (26.32)	13 (34.21)	0.876		
	No	70 (35.71)		51 (26.02)	75 (38.27)			
Coffee	<4 cups	130 (43.62)		66 (22.15)	102 (34.23)	0.237		
	≥4 cups	66 (36.07)		43 (23.5)	74 (40.44)			
Coffee frequency	Daily	88 (41.12)		53 (24.77)	73 (34.11)	0.349		
	Weekly	21 (47.73)		6 (13.64)	17 (38.64)			
	Monthly	3 (50)		0 (0)	3 (50)			
Black tea	Yes	158 (40.1)		87 (22.08)	149 (37.82)	0.375		
	No	34 (45.33)		19 (25.33)	22 (29.33)			
Caffeinated drinks	Yes	42 (30.43)		27 (19.57)	69 (50)	<0.001		
	No	102 (45.95)		55 (24.77)	65 (29.28)			
Sleep medications	Yes	7 (53.85)		1 (7.69)	5 (38.46)	0.324		
	No	117 (38.61)		75 (24.75)	111 (36.63)			
Age, years	N	206		109	180	<0.001		
	Mean	50.21		47.79	44.29			
	SD	11.06		10.82	13.55			
Height, cm	N	84		58	106	0.005		
	Mean	161.17		166.41	164.12			
	SD	9.2		10.22	9.53			
Weight, kg	N	90		59	106	0.158		
	Mean	82.4		82.12	87.28			
	SD	21.77		17.44	20.7			
BMI, kg/m^2^	N	83		58	106	0.058		
	Mean	31.56		29.68	32.36			
	SD	7.54		5.64	6.93			
HbA1c, %	N	177		91	158	0.569		
	Mean	7.98		7.82	8.09			
	SD	2.01		1.79	1.87			
Working time hours	N	53		43	95	0.538		
	Mean	6.44		6.49	6.82			
	SD	2.31		1.47	2.4			
Outdoor weekdays	N	208		111	181	0.747		
	Mean	20.93		23.48	24.81			
	SD	45.72		33.93	63.25			
Outdoor free days	N	208		111	181	0.571		
	Mean	24.34		29.28	29.81			
	SD	60.97		44.62	53.48			
Reaching work time	N	208		110	181	<0.001		
	Mean	9.16		16.06	19.05			
	SD	17.88		21.43	17.39			
Reaching home time	N	208		110	181	<0.001		
	Mean	9.36		16.15	20.43			
	SD	17.9		20.74	19.17			

## Discussion

Many studies have focused on the association between SJL and metabolic syndromes, specifically diabetes mellitus. However, we chose to look at the effect of SJL on HbA1c levels. As shown in Table 2, there was no significant association between SJL and high HbA1c levels (p=0.394), and this finding is consistent with a study done in 2013 with a sample size of 194 [11]. Per the results shown in Table 1, the prevalence of SJL in the studied population was 58.4%, while it was 69% in a study done in Europe involving 65,000 participants [4]. This could be explained by the fact that the mean age of our participants was relatively high (47.64 years) and we were focusing on diabetics primarily: the probability of having SJL decreases when you get older, and the prevalence of SJL is generally higher in the general population compared to diabetic patients. About two-thirds of people who suffer from SJL experience more than two hours of SJL in general; except in Europe, where only one-third of people with SJL has more than two hours of SJL [4]. Either very short or very long sleep duration is considered a risk factor for diabetes [9]. Our studied population had a mean sleep duration of 6.7 hours on workdays and 7.85 hours on free days, compared to a study done in Chicago with 161 participants that showed a mean sleep duration of six hours on weekdays and 6.1 hours on the weekends [14]. In a study conducted at Maastricht University involving 145 subjects, male participants had a mean sleep duration of eight hours on workdays, while it was 8.3 hours for females; on the weekends, males had 8.8 hours of sleep, while females had nine hours [2].

Most of our participants were obese, with a mean bodyweight of 84 kg. This could be attributed to the fact that we were focusing on diabetic individuals, and there is a well-known relationship between diabetes and obesity [15]. The interpretation of the results showed that those who drank caffeinated soft drinks were more likely to have SJL, and this result is supported by a previous study done on 501 participants [3]. Moreover, we found that using an alarm clock to wake up from sleep on workdays was significantly associated with SJL since it interrupted the patient's sleep. In fact, a study has suggested the following while referring to SJL: "waking on workdays is driven by an alarm clock, not the biological clock" [16]. While diabetes and sleep disorders exacerbate each other [6], we noticed that uncontrolled diabetic patients tended to drink more coffee to alleviate their daytime sleepiness [17]. This study is the first of its kind on this subject to be conducted among the Saudi population since it focuses on people's lifestyles and their correlation with many cardiovascular and metabolic diseases [2]. Hence, we believe that the findings of this study are quite significant. One strong point of our study is that we were able to arrive at substantial results despite the limited data available on this subject. As for the data, researchers collected it directly from the patients via phone calls to make sure patients fully understood the questions. We avoided alcohol-related questions since alcohol is illegal in Saudi Arabia.

Some patients reported sleeping multiple times a day, and we calculated the sum of sleep times to get the total sleep time per day for such participants. Despite our efforts, some patients did not take the questionnaire seriously; they provided either inaccurate or incomplete data. Type 2 diabetes has a high prevalence in Saudi Arabia, and 32.8% of the country's population is affected by it [6]. This will lead to many complications in the long run. However, as the results of our study show, there is no connection between SJL and HbA1c levels. We need more studies to validate these findings, which should involve carrying out a more accurate measurement of sleep duration and SJL.

## Conclusions

Our study showed that there is no significant association between SJL and HbA1c levels. The prevalence of SJL of one hour or more was 58.4% in the studied population. The predicted increase in the prevalence of diabetes mellitus in Saudi Arabia raises concerns about factors that could lead to diabetes and poor glycemic control. We recommend further studies to reach a more accurate measurement of sleep duration and SJL, and they should focus on SJL and its related complications.
